# Characteristics and Management of Patients with Substance Use Disorders Referred to a Consultation-Liaison Psychiatry Service in Lebanon

**DOI:** 10.1192/j.eurpsy.2024.758

**Published:** 2024-08-27

**Authors:** S. El Hayek, G. Kassir, H. Zalzali, K. Al Hasanieh, M. Cherro, N. Ibrahim, M. Bizri

**Affiliations:** ^1^Erada Center for Treatment and Rehabilitation in Dubai, Dubai, United Arab Emirates; ^2^American University of Beirut, Beirut, Lebanon; ^3^King’s College London, London, United Kingdom

## Abstract

**Introduction:**

Substance use disorders (SUDs) are a growing public health concern in the Arab world. To our knowledge, no previous study in Lebanon assessed the characteristics, management, and outcomes of patients with SUDs seen and managed by a consultation-liaisoin psychiatry (CLP) service.

**Objectives:**

This study explores the characteristics and management of individuals with SUDs who were referred to the CLP service in a tertiary care center in Lebanon.

**Methods:**

As part of the Consultation-Liaison at the American University of Beirut (CLAUB) analysis, we conducted a retrospective record review of patients referred to our CLP service between February 2019 and May 2020. We assessed differences between SUD and non-SUD consults using Chi-square analysis, Fisher’s exact test, or Mann-Whitney U test, as appropriate.

**Results:**

Of 1475 patients, 278 (18.8%) received a diagnosis of SUD. They were mostly males (73.7%) with an average age of 38.8 years. The most used substances were alcohol (60%) and cannabis (28.4%). Compared to non-SUD consults, patients with SUDs were more likely to be males (odds ratio OR=3.18, p<0.001) and to get intubated during admission (OR=1.81, p=0.048). Predictors of intensive care unit admission in patients with alcohol use disorder included pulmonary or endocrinological disease, benzodiazepine use disorder, and days until CLP referral.

**Conclusions:**

The results of this study highlight the high prevalence of alcohol use among individuals with SUD referred to the CLP service. Additionally, they underscore the limited treatment avenues available in this part of the world. The institution of a comprehensive CLP service is crucial to address the unmet needs of patients with SUDs who present to a general hospital setting.

**Disclosure of Interest:**

None Declared

